# Preliminary Report on Temperature Dysregulation in a Cohort of Youth with Prader–Willi Syndrome

**DOI:** 10.3390/reports8030168

**Published:** 2025-09-02

**Authors:** Daniela A. Rubin, Adam Elies, Claudia Camerino

**Affiliations:** 1 Department of Kinesiology, California State University Fullerton, 800 N. State College Blvd, Fullerton, CA 92834, USA; adam.elies@fullerton.edu; 2 Department of Precision and Regenerative Medicine, School of Medicine, University of Bari Aldo Moro, P.za 6 G. Cesare 11, 70100 Bari, Italy; ccamerino@libero.it

**Keywords:** Prader–Willi syndrome, temperature dysregulation, body composition, nervous system, oxytocin, obesity

## Abstract

**Background:** Prader–Willi Syndrome (PWS) is a genetic neurodevelopmental disorder caused by an alteration of the paternal chromosome 15q11-q13. Youth with PWS present hyperphagia, increased fat/decreased muscle mass, hypotonia, and decreased metabolic rate with risk of obesity. Thermoregulation problems have been previously reported with hypothermia in adults or hyperthermia in children/infants with PWS. **Methods:** We retrospectively examined a cohort of 44 youths with PWS, 8–16 years old, presenting with a medical history of temperature dysregulation (TD), hypothermia or hyperthermia. Participants with (n = 10) and without (n = 34) a history of TD were compared for anthropometrics, body composition, medical history, and motor characteristics. **Results:** Youth with TD presented with hypothermia (n = 8), hyperthermia (n = 2), or both conditions (n = 2). Non-parametric statistics showed no significant differences in age, anthropometrics, body composition, or motor characteristics between the groups (*p* ≥ 0.064). Those with TD presented with a higher frequency of sleep apnea versus those without (50% vs. 18%; *p* = 0.038). **Conclusions:** The prevalence of TD in the cohort was one in five youth with PWS, suggesting that the problem is not isolated. The results do not suggest that anthropometrics, body composition, or motor characteristics explain differences in temperature excursions in youths with PWS. Possible physiological mechanisms and future research are discussed.

## 1. Introduction

Prader–Willi syndrome (PWS) is a rare complex genetic disorder resulting from the lack of expression of paternally inherited genes in the chromosome 15q11-q13 region. This arises from one of three mechanisms: due to a deletion on the paternal chromosome 15, maternal uniparental disomy (UPD) with both copies of chromosome 15 inherited from the mother, or a PWS-imprinting center defect [1]. The estimated prevalence of PWS is 1:10,000 to 30,000 live births [2]. Children with PWS present with physical characteristics including almond-shaped eyes, pale skin, short stature, small hands and feet, increased fat, decreased muscle mass, as well as persistent hypotonia [3]. PWS is one of the best characterized forms of genetic obesity. Neonates with PWS present with hypotonia, poor appetite, and failure to thrive. Between 18 and 36 months of age, their caloric intake must be reduced to approximately 60–80% of the recommended childhood calories to prevent abnormal weight gain [2]. Around 4–5 years of age, they begin demonstrating an excessive interest in food, leading to the onset of overt hyperphagia manifesting at about 8 years old [4]. The characteristic hyperphagia of the syndrome, in conjunction with the decreased caloric expenditure at rest, can lead to morbid obesity unless the individual is carefully managed with behavior, diet, and exercise [2,3].

Individuals with PWS demonstrate alterations in the hypothalamic–pituitary structures in the brain, resulting in the dysregulation of many functions and hormones [5]. The lack of growth hormone production leads to the recommendation of growth hormone replacement therapy for individuals with PWS, as it aids in normalizing stature, increasing lean mass, and decreasing fat mass [6]. Therefore, the initiation of growth hormone therapy is recommended in infancy and should continue throughout childhood and adulthood [7].

Among some of the medical complications present in PWS, there are several reports of temperature dysregulation [8,9,10,11]. The issues with temperature regulation have been linked to hypothalamic involvement, autonomic nervous system (ANS) dysregulation, decreased perception of cold and warmth, and the disruption in the oxytocin signaling axis [12,13,14,15,16].

In infants, there are several case reports of hospitalizations for episodes of repeated fever and hyperthermia [8,9,10,11], as well as an early abstract presentation of five infants with recurrent hyperthermia [17]. One such case report describes three infants with PWS presenting with hypotonia and decreased sweating coupled with extremely high body temperature without identifiable infections [9]. It was speculated in this case report that hyperthermia was caused by hypothalamic dysfunction [9]. In another case, accidental hypothermia was reported in an infant with PWS where a primary disturbance in parasympathetic autonomic regulation induced the drop in body temperature to 29.8 °C [18]. Hyperpyrexia secondary to PWS was also reported in a 13-year-old boy [19].

In contrast, hypothermia is a leading cause of accidental death in 1% of the cases of adults with PWS [20]. Additionally, an early report shows some adult cases with PWS presenting with impaired thermoregulatory response to cold exposure [21]. More recently, the case of an adult with PWS was reported due to hospitalization for respiratory distress. The case subsequently developed hyperthermia and died 13 days later [14]. Similar dysregulatory conditions may be present in more individuals [16,19]; however, there is little recent documentation of this.

In this preliminary study, we determined the prevalence of temperature dysregulation (hypo- or hyperthermia) in a cohort of youth with PWS aged 8–16. Additionally, we compared demographics, body composition, medical history aspects, physical activity, and motor characteristics between those youth with and without a history of temperature dysregulation to explore possible mechanisms behind temperature dysregulation in this population.

## 2. Materials and Methods

Participants: Participants were 44 youths who participated in a home-based physical activity intervention and who had documented PWS diagnoses (protocol HSR #17-18-374 [22]. The California State University, Fullerton, Institutional Review Board reviewed and approved the use of previously collected participant data for this retrospective study under protocol HSR #24-25-217. Data collected at baseline in these study participants were included in the present study.

Medical History and Genetic Diagnoses: Parents completed a medical history questionnaire for their children and provided genetic diagnoses or hospital verification letters for their children as previously described [22]. The medical history questionnaire included questions related to their previous history of hospitalizations due to different medical reasons (including a history of hypo- and/or hyperthermia), asked about the use of growth hormone replacement therapy (GHRT), and their use of other medications. Pubertal development was determined from parents’ responses using the Pubertal Developmental Scale [23].

Anthropometrics: Standing stature was measured using a stadiometer (SECA Stadiometer, Chino, CA, USA), and body mass was measured using a calibrated digital scale (Ohaus ES Series Scale, Parsippany, NJ, USA) with the child wearing a light t-shirt and shorts. Body mass index (BMI) percentiles and z-scores were obtained from the Centers for Disease Control and Prevention website [22].

Body Composition: Determined in supine position using a dual x-ray absorptiometry scan of the full body and dual hips (GE Healthcare, Milwaukee, WI, USA) and was analyzed using the enCORE pediatric software (version 12.30.008, GE Healthcare, Milwaukee, WI, USA). Analyses provided body fat percentage, lean mass percentage, bone mineral density (BMD), and BMD z-scores.

Vitals: Measurements were taken after five minutes of seated rest, which include heart rate via telemetry (Polar Electro Inc., Bethpage, NY, USA) and blood pressure via sphygmomanometer (Welch Allyn, Skaneateles Falls, NY, USA) using standard NHANES III procedures [22].

Physical Activity: Physical activity (PA) was measured using a 4MB GT3X (Actigraph, Pensacola, FL, USA) triaxial activity monitor worn at the hip following procedures previously described [22]. Thresholds for sedentary behavior, light (LPA), moderate (MPA), and vigorous PA (VPA) were based on established cut points [24].

Motor Proficiency: motor proficiency was determined from the long form of the Bruininks-Oseretsky Test of Motor Proficiency–Version 2 (BOT-2), previously used in children with PWS, which was demonstrated to have good reliability [25]. The BOT-2 comprises 53 items and provides a total motor composite score and scores for fine motor precision and integration, manual dexterity, upper limb coordination, bilateral coordination, balance, running speed, agility, and strength.

Statistical Analyses: Descriptive statistics, including frequencies for categorical data and median, minimum, and maximum for continuous data, were computed to describe the cohort. Participants who presented with a history of hypothermia or hyperthermia were identified and assigned to the group with a history of temperature dysregulation, while participants with no history of either hypo- or hyperthermia were assigned to the group without temperature dysregulation. Groups were compared for age, anthropometrics, body composition, medical history, PA, and motor proficiency using non-parametric statistics (Mann–Whitney U test and median test for continuous variables and chi-square analyses for categorical variables). All statistics were analyzed using SPSS (version 24, IBM Corp., Armonk, NY, USA).

## 3. Results

The data of 44 children aged 8–16 years were examined for this study. Participants included 19 females and 25 males. Participants’ PWS diagnoses included deletion (45%), UPD (18%), a positive methylation test (27%), or an unidentified cause but confirmed diagnosis (10%). Most participants were in either early puberty (27%) or mid-puberty (36%). One out of five participants presented with temperature dysregulation, with the majority (80%) presenting a history of hypothermia. Demographic and participant characteristics are reported in Table 1. All statistical data regarding anthropometrics, body composition, medical history, vitals, physical activity, and motor proficiency are presented in Table 2.

### 3.1. Anthropometrics and Body Composition

There were no differences in age, height, weight, waist circumference, BMI z-scores or BMI percentiles between those with and those without temperature dysregulation, for either the distribution or the median values (*p* > 0.090 and *p* > 0.280, respectively). The frequency of youths with a healthy weight, or with overweight or obesity, was comparable across groups (χ^2^ = 0.725, *p* = 0.395). For body composition, there were also no significant differences between groups for either distribution or median values for total body fat, trunk fat, lean mass, bilateral hip BMD, bilateral hip BMD z-score, lumbar BMD, or lumbar BMD z-scores (*p* > 0.064 and *p* > 0.130, respectively).

### 3.2. Medical History and Vitals

The groups exhibited comparable vitals (resting heart rate and blood pressure) and frequency of history of hypothyroidism, diabetes type I, diabetes type II, asthma, pneumonia, sleepiness, seizures, high blood pressure, deep vein thrombosis, heart problems, varicose veins, high cholesterol, and high triglycerides (*p* > 0.086 for all). A history of sleep apnea was the only medical condition that presented at a higher frequency in those with temperature dysregulation compared to those without temperature dysregulation (50% vs. 18%, *p* = 0.038). The frequency of use of GHRT was also comparable across groups (χ^2^ = 2.105, *p* = 0.349).

### 3.3. Physical Activity and Motor Proficiency

Participants in both groups demonstrated comparable levels of sedentary activity, light intensity physical activity, and moderate-to-vigorous intensity physical activity (*p* > 0.540 and *p* > 0.921 for distribution and median differences). There were also no differences in motor proficiency between the groups (*p* > 0.102 and *p* > 0.490 for distribution and median differences).

## 4. Discussion

In this study we identified ten youths aged 8–11 years old, presenting with a history of temperature dysregulation. Eight individuals presented with a history of hypothermia (including two with a history of both hypo- and hyperthermia), and two individuals presented with only hyperthermia. The previous literature reported cases mostly in infants or young children with acute episodes of hyperthermia [8,9,10,11,17]. In adults with PWS, the literature suggests dysregulation in body temperature in PWS, mostly as hypothermia [20]. The present study shows that 23% of the youth in this cohort presented with a history of temperature dysregulation, with a majority (80%) presenting with a history of hypothermia. These youths had similar body composition characteristics, vitals, medical history (except for prevalence of sleep apnea), physical activity, and motor function parameters as those without a history of temperature dysregulation.

Temperature dysregulation in PWS has been investigated since the 1980s [21]. In an early study, it was shown that four of six adults with clinical diagnoses for PWS had difficulty with generating appropriate acute thermoregulatory responses when exposed to 4° C [21]. In contrast, the controls with non-PWS obesity elicited an increase in core body temperature in response to the cold environment [21], indicating temperature dysregulation is likely unique to PWS rather than obesity alone. No mechanistic pathway for this impaired thermoregulatory response in PWS was proposed then. The authors did discuss an overall decreased metabolic rate in PWS compared to controls with non-syndromic obesity [21], which is consistent with later studies [26].

We discuss below four possible mechanistic explanations for the temperature dysregulation reported in these ten youths with PWS and a history of temperature dysregulation. The hypothalamus, among other functions, integrates temperature homeostasis in humans and animals [27]. While the preoptic area (anterior area of the hypothalamus) contains its own heat- and cold-sensitive neurons that control regulatory responses, the posterior area receives information from peripheral receptors located in the skin as well as core receptors located in the spinal cord and abdominal and thoracic areas. The posterior hypothalamus integrates information from all receptors and connects to the ANS to generate temperature-increasing or temperature-decreasing responses [28]. One possibility is that in PWS, there is a dysregulation in the integration of peripheral thermal receptors’ afferent input and the subsequent efferent responses. In individuals without syndromic disorders, exposure to a cold environment elicits compensatory mechanisms to increase or maintain the core temperature, such as vasoconstriction, shivering, or non-shivering thermogenesis or movement [29]. Animal studies have suggested that the origin of the altered thermal perception in PWS might be related to a reduction in sensory neurons at the level of dorsal root ganglia [30]. Additionally, lower and higher thresholds for cold or warm sensations in the palms of hands and feet in adults with PWS have been shown with no evident alteration in peripheral nerve conduction [15]. While these results are somewhat inconclusive, they suggest that when individuals with PWS are exposed to much more extreme environmental temperatures, they could exhibit greater temperature excursions compared to those without PWS, as they may not exhibit compensatory mechanisms because of different thresholds.

Dysfunction in the sympathetic nervous system is a second possible explanation for temperature dysregulation in PWS [12], as it has also been suggested in Schaaf–Yang syndrome [31]. Schaaf–Yang syndrome is a rare neurodevelopmental syndrome caused by variants in the melanoma antigen L2 (MAGEL) gene located in the PWS critical region 15q11-15q13 [32]. Prolonged hyperthermia of unidentified cause was reported in a 1-year-old patient with Schaaf–Yang syndrome [31]. In response to increases in body temperature, the preoptic area of the hypothalamus, through the sympathetic nervous system cholinergic fibers, triggers heat dissipation responses such as sweating, while adrenergic fibers will trigger vasodilation [28]. To date, one experiment attempted to evaluate sweating responses in children with PWS to assess impaired sympathetic nervous system activation [33]. However, the results were inconclusive because data were lacking in most participants [33]. Though if there were impaired sympathetic nervous system responses, such as sweating or skin vasodilation, this could lead to heat accumulation in the core and presentation of hyperthermia. In our participants, there are no differences in resting vitals between the groups.

A third potential mechanism that has been hypothesized involves a dysregulation in the hypothalamic hormone oxytocin [13,34]. Post-mortem studies show that adults with PWS present a reduced volume of the paraventricular nucleus (PVN) in the anterior hypothalamus with fewer oxytocin-releasing neurons and lower mRNA oxytocin expression [35,36]. Interestingly, concentrations of oxytocin in the cerebrospinal fluid of five adults with PWS were higher than in six participants without PWS [37]. Moreover, children with PWS showed elevated circulating blood oxytocin compared to their healthy-weight siblings [38]. However, normal ranges of circulating oxytocin have also been shown in children and adults with PWS [39], as well as no differences in circulating plasma or saliva oxytocin in both adolescents and adults with PWS compared to controls [40].

Genetic animal models of oxytocin deficiency or oxytocin receptor deficiency demonstrate a reduced sympathetic tone and a reduced capacity for heat production [41,42]. In wild-type mice, oxytocin regulates thermogenesis in the skeletal muscle during acute cold exposure by potentiating shivering through the contraction of slow-twitch muscle fibers [43]. Wild-type mice exposed to a cold environment for five days show increased expression of the oxytocin receptors in the PVN and supraoptic nucleus but decreased circulating oxytocin, potentially due to a negative feedback loop between the hypothalamus and circulating oxytocin [43]. Conversely, in individuals with PWS, the disruption of this negative feedback loop may result in decreased expression of oxytocin-releasing neurons in the brain [35] and higher circulating oxytocin [37,38]. Hence, in individuals with PWS, the increased circulating oxytocin may in turn make slow-twitch muscle fibers less responsive to the oxytocin’s signal, causing a decreased muscle tone [13]. Consistent with this hypothesis, adults with PWS showed reduced serum concentrations of Cyclic ADP–Ribose Hydrolase 1 (CD38) and Growth/Differentiation Factor 8/Myostatin. These are related to dysregulation in oxytocin release and neuromuscular impairment, respectively [44].

Last, excess fatness has been implicated with higher core temperatures throughout the day in individuals with obesity but without PWS [45], showing specific thermoregulatory responses during exercise [46]. Our results do not suggest that body composition (fat, lean, and bone) explains differences in presenting with or without a history of temperature dysregulation. Hence, based on the results of this study, it appears that body temperature dysregulation is unrelated to body composition in youth with PWS.

Youths in this study achieved about ½ of the recommended 60 min of moderate to vigorous PA, as previously found [47]. This is of concern as PA contributes to better body composition and motor proficiency in those with PWS, as in children with neurotypical development [48,49]. In this regard, it is important to highlight that children exhibit a lower sweat rate and a higher core threshold for core temperature to trigger a compensatory sweating response compared to adults [50]. However, children with neurotypical development exhibit thermoregulatory mechanisms that allow them to cope with exercise-induced increases in body temperature, such as a higher body surface area to mass ratio and greater skin blood flow than adults, which allows for heat dissipation [50,51]. When one considers the important role of exercise in the health of a young person with PWS [2], understanding potential temperature regulation issues in PWS becomes important. Future studies could consider measurements or estimates of core temperature to detect the presence or lack thereof of temperature excursions over a time frame representative of habitual activity. Additionally, evaluating thermoregulatory responses to acute exercise may also be important, specifically as exercise is crucial in the management of body weight in PWS.

In this report we identified 10 youths from a cohort of 44 youths with PWS whose parents indicated in their medical history issues with temperature regulation (hyperthermia, hypothermia, or both) that did not result in hospitalization. Only the prevalence of sleep apnea was significantly higher in those with temperature dysregulation compared to those without. It is possible these two conditions could be co-occurring or be related, as there were no actual measurements of body temperature, meaning the parents’ reports could not be empirically verified. This is a main limitation since individuals may provide different perceptions of temperature fluctuation in their children. Additionally, there was no contextual information for the history of hypothermia, hyperthermia, or sleep apnea. There was no information as to whether the parent measured the body temperature, whether this body temperature was high or low, was “usual”, or whether it was an episode of high or low temperature, and the duration of each such episode. Additionally, there was no other information that could have further explained the dysregulation, such as ambient conditions, exercise, or hydration. PWS molecular subclasses were not available for all subjects to conduct a comparison between the groups for the role of molecular diagnoses. Moreover, for many of the medical history variables compared between the groups, there were not enough cases per cell (a minimum of five), which limits the power to detect the differences between the groups. Last, there were only ten cases that presented temperature dysregulation, and hence, the sample size for that group was small, which means that the findings are preliminary.

## 5. Conclusions

This is a report of multiple youth with PWS with a history of hypo- or hyperthermia. These data suggest that these temperature excursions are not isolated cases as described in previous case reports [8,9,11,14,18,19]. Future research is needed to document these temperature excursions using esophageal pills to measure core temperature over the course of several days. Additionally, research is needed to elucidate the origins of these temperature fluctuations, including the measurement of oxytocin, ANS involvement, as well as potentially confounding medical complications or comparisons with youth without PWS with and without obesity. Last, evaluation of temperature regulation capabilities during exercise or in response to heat or cold exposure can also provide insight into the problem.

## Figures and Tables

**Table 1:** Cohort characteristics presented as frequencies or minimum–maximum values.

Participant Characteristic	Data Values
Demographics	
Age (Years)	8–16
Sex (Females/Males)	19/25
Number of Participants	44
Cases w/Temperature Dysregulation	10
Cases w/o Temperature Dysregulation	34
Ethnicity	
Caucasian	27
Hispanic	12
Asian	3
African American	1
Pubertal Status	
Pre-Pubertal	6
Early-pubertal	12
Mid-pubertal	16
Late-pubertal	7
PWS Subtype	
Deletion	20
Uniparental Disomy	8
DNA Methylation +	12
Unidentified	4
Type of Dysregulation	
Hypothermia	8
Hyperthermia	4
Both	2

Key: PWS = Prader–Willi Syndrome.

**Table 2:** Participant characteristics by groups presenting with or without temperature dysregulation presented as frequencies or medians with minimum–maximum values.

Participants	Temperature Dysregulation	No Temperature Dysregulation	*p*-Value of the Distribution	*p*-Value of the Median	*p*-Value of the Frequency
Sample Size	N = 10	N = 34	-	-	-
Demographics					
Age (Years)	11.5 (8.0–14.0)	9.0 (8.0–16.0)	*p* = 0.227	*p* = 0.765	-
Anthropometrics					
Height (cm)	154.5 (125.9–160.7)	136.3 (125.0–178.4)	*p* = 0.090	*p* = 0.280	-
Weight (kg)	59.8 (25.7–90.1)	51.9 (27.1–130.3)	*p* = 0.689	*p* = 0.719	-
Waist Circumference (cm)	88.9 (60.3–123.0)	84.0 (60.4–141.0)	*p* = 0.901	*p* = 0.844	-
BMI (Z-Score)	1.59 ([−0.50]–2.76)	2.09 ([−0.24]–3.01)	*p* = 0.312	*p* = 0.719	-
BMI Percentile (%)	93.5 (30.0–99.0)	97.5 (40.0–100.0)	*p* = 0.286	*p* = 0.359	-
Weight Status					*p* = 0.395
Healthy weight	N = 3	N = 6	-	-	-
Overweight/Obese	N = 7	N = 28	-	-	-
Body Composition					
Total Body Fat (%)	42.80 (31.90–58.50)	48.35 (17.90–60.80)	*p* = 0.534	*p* = 0.719	-
Trunk fat (%)	40.55 (29.00–59.60)	47.80 (17.40–60.60)	*p* = 0.368	*p* = 0.280	-
Lean Mass (kg)	31.0 (16.5–37.4)	24.2 (18.2–55.6)	*p* = 0.261	*p* = 0.280	-
Bilateral hip BMD (g/cm^2^)	0.80 (0.49–0.97)	0.74 (0.61–1.34)	*p* = 0.945	*p* = 0.719	-
Lumbar spine BMD (g/cm^2^)	0.8790 (0.76–1.06)	0.91 (0.75–1.54)	*p* = 0.566	*p* = 1.000	-
Bilateral hip BMD (Z-Score)	−1.10 ([−2.80]–1.10)	−0.20 ([−1.90]–2.30)	*p* = 0.108	*p* = 0.844	-
Lumbar spine BMD (Z-Score)	−0.10 ([−1.30]–4.10)	1.00 ([−0.50]–4.10)	*p* = 0.064	*p* = 0.130	-
Vitals					
Heart rate (beat/min)	80 (64–101)	80 (56–131)	*p* = 0.913	*p* = 0.876	
Systolic BP (mmHg)	108 (82–127)	102 (74–126)	*p* = 0.432	*p* = 0.600	
Diastolic BP (mmHg)	65 (56–76)	64 (44–88)	*p* = 0.913	*p* = 0.931	
History Of:					
Hypothyroidism	N = 2	N = 6	-	-	*p* = 0.865
Diabetes type I	N = 1	N = 2	-	-	*p* = 0.668
Diabetes type II	N = 1	N = 1	-	-	*p* = 0.359
Asthma	N = 5	N = 9	-	-	*p* = 0.160
Sleep Apnea	N = 5	N = 6	-	-	*p* = 0.038 *
Pneumonia	N = 3	N = 3	-	-	*p* = 0.086
Excessive Sleepiness	N = 4	N = 12	-	-	*p* = 0.786
Seizures	N = 4	N = 6	-	-	*p* = 0.138
High Blood Pressure	N = 0	N = 0	-	-	N/A
Deep Vein Thrombosis	N = 0	N = 0	-	-	N/A
Heart Problems	N = 0	N = 1	-	-	*p* = 0.583
Varicose Veins	N = 1	N = 1	-	-	*p* = 0.346
High Cholesterol	N = 0	N = 4	-	-	*p* = 0.255
High Triglycerides	N = 0	N = 3	-	-	*p* = 0.331
GHRT Usage					*p* = 0.349
Current Only	N = 9	N = 23	-	-	-
Past Only	N = 1	N = 8	-	-	-
Never	N = 0	N = 3	-	-	-
Physical Activity					
Sedentary (min/day)	668.2 (497.2–775.0)	660.3 (414.3–780.7)	*p* = 0.540	*p* = 0.921	-
Light PA (min/day)	117.0 (87.8–255.3)	126.2 (75.1–210.2)	*p* = 0.897	*p* = 0.921	-
Moderate-Vigorous PA (min/day)	33.4 (13.8–54.2)	26.2 (2.9–87.8)	*p* = 0.725	*p* = 0.921	-
Motor Proficiency (Arbitrary Units)	31.0 (20.0–34.0)	25.5 (20.0–34.0)	*p* = 0.102	*p* = 0.490	-

Key: BMI = Body-Mass-Index; BMD = Bone Mineral Density; BP = Blood Pressure; GHRT = Growth Hormone Replacement Therapy; PA = Physical Activity. Significant values (*p* ≤ 0.05) are marked with *.

## Data Availability

The data that support the findings of this study are presented in the manuscript, further inquiries should be directed to the corresponding author [D.A.R.].

## Abbreviations

The following abbreviations are used in this manuscript:

PWS	Prader–Willi Syndrome
TD	Temperature Dysregulation
UPD	Uniparental Disomy
ANS	Autonomic Nervous System
GHRT	Growth Hormone Replacement Therapy
BMI	Body Mass Index
BMD	Bone Mineral Density
PA	Physical Activity
LPA	Light Physical Activity
MPA	Moderate Physical Activity
VPA	Vigorous Physical Activity
PVN	Paraventricular Nucleus

## References

[1] Butler M.G., Hartin S.N., Hossain W.A., Manzardo A.M., Kimonis V., Dykens E. (2019). Molecular genetic classification in Prader-Willi syndrome: A multisite cohort study. J. Med. Genet..

[2] Driscoll D.J., Miller J.L., Cassidy S.B., Adam M.P., Feldman J., Mirzaa G.M. (1993). Prader-Willi Syndrome. GeneReviews^®^ [Internet].

[3] Butler M.G., Miller J.L., Forster J.L. (2019). Prader-Willi Syndrome—Clinical Genetics, Diagnosis and Treatment Approaches: An Update. Curr. Pediatr. Rev..

[4] Miller J.L., Lynn C.H., Driscoll D.C., Goldstone A.P., Gold J.A., Kimonis V. (2011). Nutritional phases in Prader-Willi syndrome. Am. J. Med. Genet. A.

[5] Madeo S.F., Zagaroli L., Vandelli S., Calcaterra V., Crino A., De Sanctis L. (2024). Endocrine features of Prader-Willi syndrome: A narrative review focusing on genotype-phenotype correlation. Front. Endocrinol..

[6] Angulo M., Abuzzahab M.J., Pietropoli A., Ostrow V., Kelepouris N., Tauber M. (2020). Outcomes in children treated with growth hormone for Prader-Willi syndrome: Data from the ANSWER Program(R) and NordiNet(R) International Outcome Study. Int. J. Pediatr. Endocrinol..

[7] Hoybye C., Holland A.J., Driscoll D.J., Clinical and Scientific Advisory Board of The International Prader-Willi Syndrome Organisation (2021). Time for a general approval of growth hormone treatment in adults with Prader-Willi syndrome. Orphanet J. Rare Dis..

[8] Donoso A., Arriagada D., Campbell S., Cruces P. (2013). Multiorgan failure associated with hyperthermia in an infant with Prader-Willi syndrome. case report. Arch. Argent Pediatr..

[9] Ince E., Ciftci E., Tekin M., Kendirli T., Tutar E., Dalgic N. (2005). Characteristics of hyperthermia and its complications in patients with Prader Willi syndrome. Pediatr. Int..

[10] Landau D., Hirsch H.J., Gross-Tsur V. (2016). Case report: Severe asymptomatic hyponatremia in Prader-Willi Syndrome. BMC Pediatr..

[11] Yalcin S.S., Kale E., Topaloglu H., Tuncbilek E. (2007). A hypotonic infant with tachycardia and fever of unknown origin. J. Pediatr. Health Care.

[12] Butler M.G., Victor A.K., Reiter L.T. (2023). Autonomic nervous system dysfunction in Prader-Willi syndrome. Clin. Auton. Res..

[13] Camerino C. (2023). Oxytocin’s Regulation of Thermogenesis May Be the Link to Prader-Willi Syndrome. Curr. Issues Mol. Biol..

[14] Vahil N., Albrektson K., Dean J. (2023). Acute Non-exertional Hyperthermia With Multi-organ Failure in an Adult With Prader-willi Syndrome: A Case Report. Am. J. Respir. Crit. Care Med..

[15] Priano L., Miscio G., Grugni G., Milano E., Baudo S., Sellitti L. (2009). On the origin of sensory impairment and altered pain perception in Prader-Willi syndrome: A neurophysiological study. Eur. J. Pain.

[16] Williams M.S., Rooney B.L., Williams J., Josephson K., Pauli R. (1994). Investigation of thermoregulatory characteristics in patients with Prader-Willi syndrome. Am. J. Med. Genet..

[17] Wise M., Zoghbi H., Edwards M., Byrd L., Guttmacher A., Greenberg F. (1991). Hyperthermia in infants with Prader-Willi syndrome (abstract). Am. J. Med. Genet..

[18] Watanabe T., Iwabuchi H., Oishi M. (2003). Accidental hypothermia in an infant with Prader-Willi syndrome. Eur. J. Pediatr..

[19] McVea S., Thompson A.J., Abid N., Richardson J. (2016). Thermal dysregulation in Prader-Willi syndrome: A potentially fatal complication in adolescence, not just in infancy. BMJ Case Rep..

[20] Butler M.G., Manzardo A.M., Heinemann J., Loker C., Loker J. (2017). Causes of death in Prader-Willi syndrome: Prader-Willi Syndrome Association (USA) 40-year mortality survey. Genet. Med..

[21] Bray G.A., Dahms W.T., Swerdloff R.S., Fiser R.H., Atkinson R.L., Carrel R.E. (1983). The Prader-Willi syndrome: A study of 40 patients and a review of the literature. Medicine.

[22] Rubin D.A., Wilson K.S., Castner D.M., Dumont-Driscoll M.C. (2019). Changes in Health-Related Outcomes in Youth With Obesity in Response to a Home-Based Parent-Led Physical Activity Program. J. Adolesc. Health.

[23] Petersen A.C., Crockett L., Richards M. (1988). A self-report measure of pubertal status: Reliability, validity, and initial norms. J. Youth Adolesc..

[24] Evenson K.R., Catellier D.J., Gill K., Ondrak K.S., McMurray R.G. (2008). Calibration of two objective measures of physical activity for children. J. Sports Sci..

[25] Lam M.Y., Rubin D.A., White E., Duran A.T., Rose D.J. (2018). Test-retest reliability of the Bruininks-Oseretsky Test of Motor Proficiency, Second Edition for youth with Prader-Willi syndrome. Ann. Phys. Rehabil. Med..

[26] Butler M.G., Theodoro M.F., Bittel D.C., Donnelly J.E. (2007). Energy expenditure and physical activity in Prader-Willi syndrome: Comparison with obese subjects. Am. J. Med. Genet. A.

[27] Tan C.L., Knight Z.A. (2018). Regulation of Body Temperature by the Nervous System. Neuron.

[28] Guyton A.C., Hall J.E. (2016). Textbook of Medical Physiology.

[29] Fernandez-Pena C., Reimundez A., Viana F., Arce V.M., Senaris R. (2023). Sex differences in thermoregulation in mammals: Implications for energy homeostasis. Front. Endocrinol..

[30] Kuwako K., Hosokawa A., Nishimura I., Uetsuki T., Yamada M., Nada S. (2005). Disruption of the paternal necdin gene diminishes TrkA signaling for sensory neuron survival. J. Neurosci..

[31] Poomehr P., Behzadi A., Farahbakhsh N., Bahadori A. (2025). Schaaf-Yang Syndrome Presenting with Prolonged Hyperthermia in a Child: A Case Report. Arch. Pediatr. Infect. Dis..

[32] Schubert T., Schaaf C.P. (2025). MAGEL2 (patho-) physiology and Schaaf-Yang syndrome. Dev. Med. Child Neurol..

[33] Richer L.P., Tan Q., Butler M.G., Avedzi H.M., DeLorey D.S., Peng Y. (2023). Evaluation of Autonomic Nervous System Dysfunction in Childhood Obesity and Prader-Willi Syndrome. Int. J. Mol. Sci..

[34] Camerino C. (2024). The Pivotal Role of Oxytocin’s Mechanism of Thermoregulation in Prader-Willi Syndrome, Schaaf-Yang Syndrome, and Autism Spectrum Disorder. Int. J. Mol. Sci..

[35] Swaab D.F., Purba J.S., Hofman M.A. (1995). Alterations in the hypothalamic paraventricular nucleus and its oxytocin neurons (putative satiety cells) in Prader-Willi syndrome: A study of five cases. J. Clin. Endocrinol. Metab..

[36] Bochukova E.G., Lawler K., Croizier S., Keogh J.M., Patel N., Strohbehn G. (2018). A Transcriptomic Signature of the Hypothalamic Response to Fasting and BDNF Deficiency in Prader-Willi Syndrome. Cell Rep..

[37] Martin A., State M., Anderson G.M., Kaye W.M., Hanchett J.M., McConaha C.W. (1998). Cerebrospinal fluid levels of oxytocin in Prader-Willi syndrome: A preliminary report. Biol. Psychiatry.

[38] Johnson L., Manzardo A.M., Miller J.L., Driscoll D.J., Butler M.G. (2016). Elevated plasma oxytocin levels in children with Prader-Willi syndrome compared with healthy unrelated siblings. Am. J. Med. Genet. A.

[39] Hoybye C., Barkeling B., Espelund U., Petersson M., Thoren M. (2003). Peptides associated with hyperphagia in adults with Prader-Willi syndrome before and during GH treatment. Growth Horm. IGF Res..

[40] Rice L.J., Agu J., Carter C.S., Harris J.C., Nazarloo H.P., Naanai H. (2023). The relationship between endogenous oxytocin and vasopressin levels and the Prader-Willi syndrome behaviour phenotype. Front. Endocrinol..

[41] Takayanagi Y., Kasahara Y., Onaka T., Takahashi N., Kawada T., Nishimori K. (2008). Oxytocin receptor-deficient mice developed late-onset obesity. Neuroreport.

[42] Camerino C. (2009). Low sympathetic tone and obese phenotype in oxytocin-deficient mice. Obesity.

[43] Conte E., Romano A., De Bellis M., De Ceglia M., Rosaria-Carratu M., Gaetani S. (2021). Oxtr/TRPV1 expression and acclimation of skeletal muscle to cold-stress in male mice. J. Endocrinol..

[44] Pascut D., Giraudi P.J., Banfi C., Ghilardi S., Tiribelli C., Bondesan A. (2024). Characterization of Circulating Protein Profiles in Individuals with Prader-Willi Syndrome and Individuals with Non-Syndromic Obesity. J. Clin. Med..

[45] Hoffmann M.E., Rodriguez S.M., Zeiss D.M., Wachsberg K.N., Kushner R.F., Landsberg L. (2012). 24-h core temperature in obese and lean men and women. Obesity.

[46] Wijers S.L., Saris W.H., Van Marken Lichtenbelt W.D. (2010). Cold-induced adaptive thermogenesis in lean and obese. Obesity.

[47] Castner D.M., Tucker J.M., Wilson K.S., Rubin D.A. (2014). Patterns of habitual physical activity in youth with and without Prader-Willi Syndrome. Res. Dev. Disabil..

[48] Rubin D.A., Wilson K.S., Orsso C.E., Gertz E.R., Haqq A.M., Castner D.M. (2020). A 24-Week Physical Activity Intervention Increases Bone Mineral Content without Changes in Bone Markers in Youth with PWS. Genes.

[49] Rubin D.A., Wilson K.S., Dumont-Driscoll M., Rose D.J. (2019). Effectiveness of a Parent-led Physical Activity Intervention in Youth with Obesity. Med. Sci. Sports Exerc..

[50] Falk B., Dotan R. (2008). Children’s thermoregulation during exercise in the heat: A revisit. Appl. Physiol. Nutr. Metab..

[51] Notley S.R., Akerman A.P., Meade R.D., McGarr G.W., Kenny G.P. (2020). Exercise Thermoregulation in Prepubertal Children: A Brief Methodological Review. Med. Sci. Sports Exerc..

